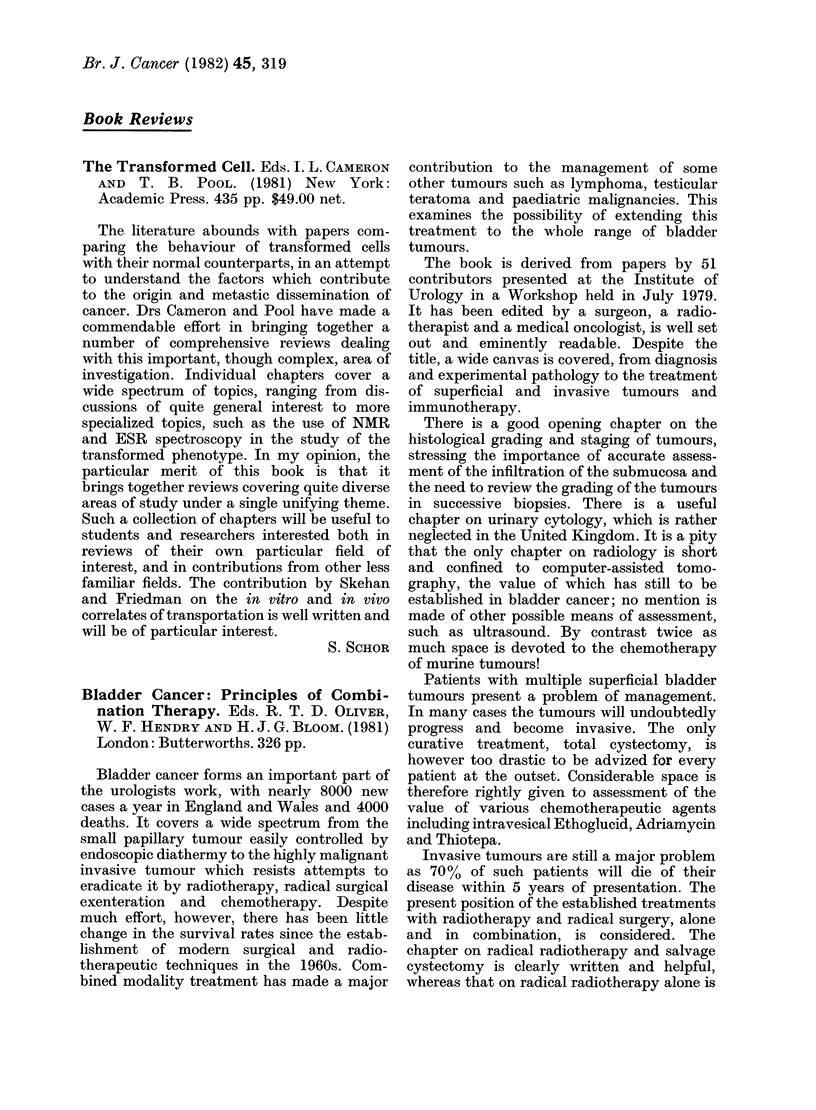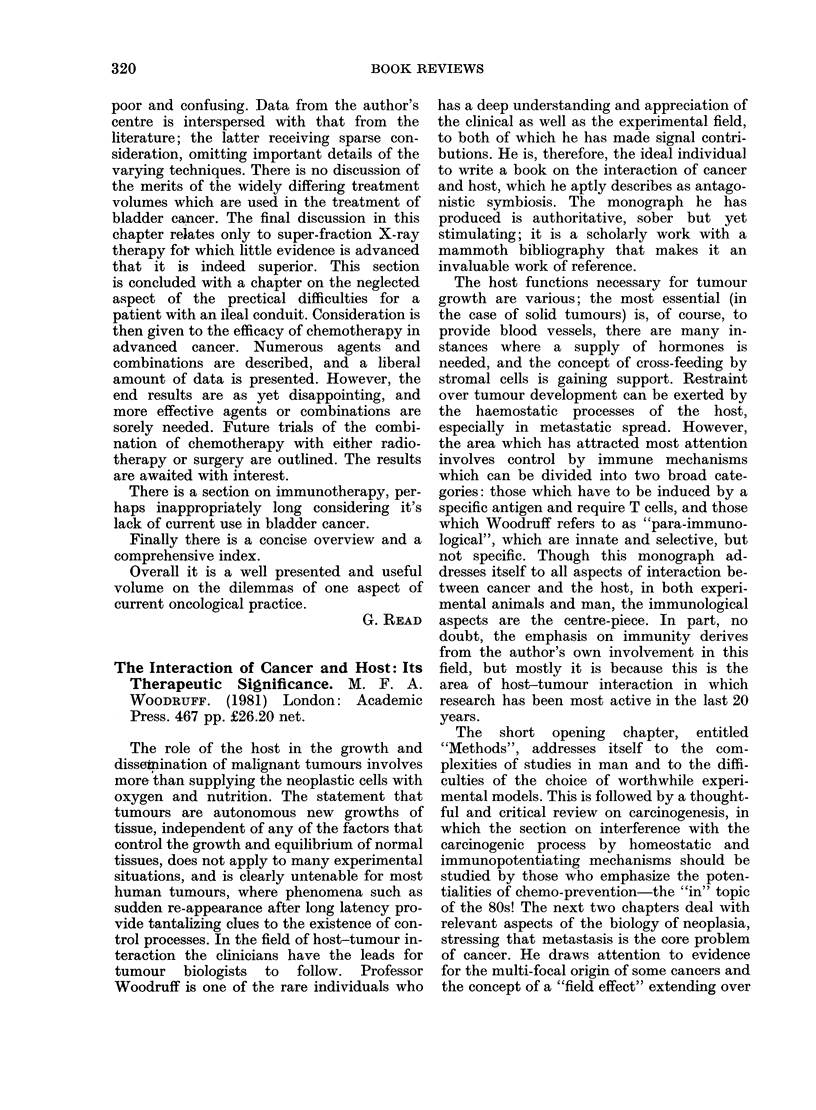# Bladder Cancer: Principles of Combination Therapy

**Published:** 1982-02

**Authors:** G. Read


**Bladder Cancer: Principles of Combination Therapy.** Eds. R. T. D. Oliver, W. F. Hendry and H. J. G. Bloom. (1981) London: Butterworths. 326 pp.

Bladder cancer forms an important part of the urologists work, with nearly 8000 new cases a year in England and Wales and 4000 deaths. It covers a wide spectrum from the small papillary tumour easily controlled by endoscopic diathermy to the highly malignant invasive tumour which resists attempts to eradicate it by radiotherapy, radical surgical exenteration and chemotherapy. Despite much effort, however, there has been little change in the survival rates since the establishment of modern surgical and radiotherapeutic techniques in the 1960s. Combined modality treatment has made a major contribution to the management of some other tumours such as lymphoma, testicular teratoma and paediatric malignancies. This examines the possibility of extending this treatment to the whole range of bladder tumours.

The book is derived from papers by 51 contributors presented at the Institute of Urology in a Workshop held in July 1979. It has been edited by a surgeon, a radiotherapist and a medical oncologist, is well set out and eminently readable. Despite the title, a wide canvas is covered, from diagnosis and experimental pathology to the treatment of superficial and invasive tumours and immunotherapy.

There is a good opening chapter on the histological grading and staging of tumours, stressing the importance of accurate assessment of the infiltration of the submucosa and the need to review the grading of the tumours in successive biopsies. There is a useful chapter on urinary cytology, which is rather neglected in the United Kingdom. It is a pity that the only chapter on radiology is short and confined to computer-assisted tomography, the value of which has still to be established in bladder cancer; no mention is made of other possible means of assessment, such as ultrasound. By contrast twice as much space is devoted to the chemotherapy of murine tumours!

Patients with multiple superficial bladder tumours present a problem of management. In many cases the tumours will undoubtedly progress and become invasive. The only curative treatment, total cystectomy, is however too drastic to be advized for every patient at the outset. Considerable space is therefore rightly given to assessment of the value of various chemotherapeutic agents including intravesical Ethoglucid, Adriamycin and Thiotepa.

Invasive tumours are still a major problem as 70% of such patients will die of their disease within 5 years of presentation. The present position of the established treatments with radiotherapy and radical surgery, alone and in combination, is considered. The chapter on radical radiotherapy and salvage cystectomy is clearly written and helpful, whereas that on radical radiotherapy alone is poor and confusing. Data from the author's centre is interspersed with that from the literature; the latter receiving sparse consideration, omitting important details of the varying techniques. There is no discussion of the merits of the widely differing treatment volumes which are used in the treatment of bladder cancer. The final discussion in this chapter relates only to super-fraction X-ray therapy for which little evidence is advanced that it is indeed superior. This section is concluded with a chapter on the neglected aspect of the prectical difficulties for a patient with an ileal conduit. Consideration is then given to the efficacy of chemotherapy in advanced cancer. Numerous agents and combinations are described, and a liberal amount of data is presented. However, the end results are as yet disappointing, and more effective agents or combinations are sorely needed. Future trials of the combination of chemotherapy with either radiotherapy or surgery are outlined. The results are awaited with interest.

There is a section on immunotherapy, perhaps inappropriately long considering it's lack of current use in bladder cancer.

Finally there is a concise overview and a comprehensive index.

Overall it is a well presented and useful volume on the dilemmas of one aspect of current oncological practice.

G. Read